# Designing and psychometric assessment of the scale of factors influencing HPV vaccine uptake behaviors in young adults

**DOI:** 10.1186/s13027-022-00461-z

**Published:** 2022-09-10

**Authors:** Soudabeh Yarmohammadi, Mohtasham Ghaffari, Yadollah Mehrabi, Samira Mousavi, Ali Ramezankhani

**Affiliations:** 1 School of Public Health and Safety, Shahid Beheshti University of Medical Sciences, Tehran, Iran; 2 Environmental and Occupational Hazards Control Research Centre, School of Public Health, Shahid Beheshti University of Medical Sciences, Tehran, Iran; 3 Department of Epidemiology, School of Public Health and Safety, Shahid Beheshti University of Medical Sciences, Tehran, Iran; 4 Department of Epidemiology and Biostatistics, Faculty of Health, Isfahan University of Medical Sciences, Isfahan, Iran; 5 Department of Public Health, School of Health and Safety, Shahid Beheshti University of Medical Sciences, Tehran, Iran

**Keywords:** Effective factors, Papillomavirus vaccines, Young adults, Psychometrics, Validation, Scale

## Abstract

**Background:**

In order to increase HPV vaccination, it is necessary to identify the factors influencing vaccination behavior among different cultures and the young adult populations. To evaluate the factors influencing HPV vaccine uptake behaviors, valid, reliable, and culture-compatible scales are required. This study was conducted with the aim of designing and psychometric assessment of the scale of factors influencing HPV vaccine uptake behaviors in Young Adults (FI(HPV)VUBYA) in Iran.

**Methods:**

The present study was carried out in a mixed-method in two steps: (a) Generating items using a qualitative study and literature review and (b) Reducing items by psychometric assessment of the designed scale. The initial set of items (N = 80) was prepared based on a qualitative study and literature review. A total of 400 young adults participated in online data collection from November 2019 to February 2020. The validity (face, content, and construct) and reliability (internal consistency and stability) of the scale were evaluated.

**Results:**

The exploratory factor analysis (EFA) revealed that the scale has 7 factors, explaining 57.84% of the total extracted variance. There was also a knowledge factor that EFA did not analyze, but its validity and reliability were evaluated with 7 other factors. The results of confirmatory factor analysis showed a good model fit. Convergent and divergent validity of the scale was accepted for all factors. Good reliability was also reported for the scale.

**Conclusion:**

FI(HPV)VUBYA 8-factor scale has good validity and reliability among young Iranian adults. Due to its appropriate psychometric properties, this scale can be used on this population in future studies.

## Background

Cervical cancer is a serious public health problem and the fourth leading cause of death in women due to cancer, with a mortality rate of 7.5% [1, 2]. Approximately, 270,000 deaths from cervical cancer occurred in 2015 in low- and middle-income countries, which was 18 times more than in developed countries [3]. There is a relationship between the incidence of cervical cancer and infection with the human papillomavirus (HPV) [4]. HPV is one of the most common sexually transmitted diseases [5]. Persistent infections with high-risk types of HPV, especially strains 16 and 18, are involved in 20% and 50% of cervical cancer, respectively [6].

The HPV vaccine is an effective way to reduce the risk of HPV transmission in both men and women [7]. In the review of the safety of the HPV vaccine, the Centers for Disease Control and Prevention reported no difference in side effects between vaccinated and unvaccinated individuals [8]. In 2006, the HPV vaccine was approved by the US Food and Drug Administration for adolescent girls [9] and in 2011, the HPV 9-valent vaccine for boys [10]. However, HPV vaccines are less common in developing countries [11, 12]. For instance, HPV vaccinations among Vietnamese youth (5.7%) were significantly lower than their American peers (42%) [13]. In a study by Dadashi et al. in Iran, the prevalence of HPV infections (38.6%) was reported in women with cervical cancer [14]. The estimated cost of HPV vaccination in Iran was economically viable [15]. The HPV-related vaccines are rare in Iran and we do not have statistics on the rate of HPV vaccination.

Significant demographic and social changes have taken place worldwide [16], including increasing the age of marriage, increasing the educated people, and changing the family structure [17]. As a result, premarital sex has gradually become very common among young people in the world [18]. In young adults aged 15–24 years, unsafe sex plays an important role in the disability-adjusted life years [19]. Focusing on this age group is important because young adults are at higher risk for HPV infection [20].

Despite global changes in the liberalization in attitudes toward premarital sex and the effects of the media and globalization, Iranian youth face significant paradoxes due to the cultural and Islamic background of society. In addition, current Iranian society faces disagreements about sexual attitudes [21]. As a result, sexual issues are not as openly expressed or discussed as in Western countries [22]. Given that 48% of the Iranian population is between the ages of 15 and 24 years [23], this can increase sexually transmitted diseases among young adults in Iran.

Despite the importance of studying the FI(HPV)VUBYA, quantitative and qualitative studies have identified these factors. They include lack of information about the vaccine, its side effects, lack of advice from healthcare providers about the vaccine, vaccine costs, norms and social values related to sexual activity, and lack of trust in vaccination programs and healthcare providers [24–27]. In Iran, there is no study examining FI(HPV)VUBYA based on a qualitative study, and there are only limited cross-sectional studies on the attitudes and knowledge of women, parents, youth, and healthcare providers about HPV and related vaccines. In these studies, it has been reported that people did not get the vaccine due to lack of knowledge about HPV and related vaccines [28–30]. One of the biggest concerns about the HPV vaccine has been reported its side effects and its costs, respectively [31].

Questionnaires were designed to inform parents and specialists about the HPV vaccine. A questionnaire was designed in Malaysia to assess the knowledge, attitude, and practice of adults about HPV vaccination from the perspective of healthcare providers [32]. A questionnaire was also designed to increase the HPV vaccine completion in young adults by surveying students about the HPV [33]. Another questionnaire was designed in France to determine the doubt about HPV vaccination among mothers [34]. A questionnaire was also designed to determine the intention of getting vaccines for six diseases (measles, pertussis, pneumococcal infection, influenza, tetanus, and HPV) from the perspective of specialists [35]. Another questionnaire was designed in Israel to assess the knowledge, attitude, and practice of HPV vaccination from the perspective of physicians [36]. Most of the designed questionnaires examined the viewpoints of mothers and experts about vaccination or completing their vaccination period in young people, and also obtained the items of the questionnaire from the literature review. No study or limited studies were found examining the FI(HPV)VUBYA questionnaire from a young adult’s perspective and used a qualitative study to design items.

Regardless of the quality of the questionnaires, differences in the target population and the lack of a complete and valid questionnaire examining all aspects of FI(HPV)VUBYA was the reason for not using these questionnaires in the study population. Given the importance of FI(HPV)VUBYA as an indicator to improve vaccination and the need to pay more attention to these factors for young adults, this study aims to identify FI(HPV)VUBYA and then design a valid scale to accurately measure these factors. The FI(HPV)VUBYA results will have benefits for both young adults and the health system. This helps identify barriers to vaccination from a variety of perspectives, and the health system addresses them with the help of universities, schools, and health centers.

## Designing

**Fig. 1:**
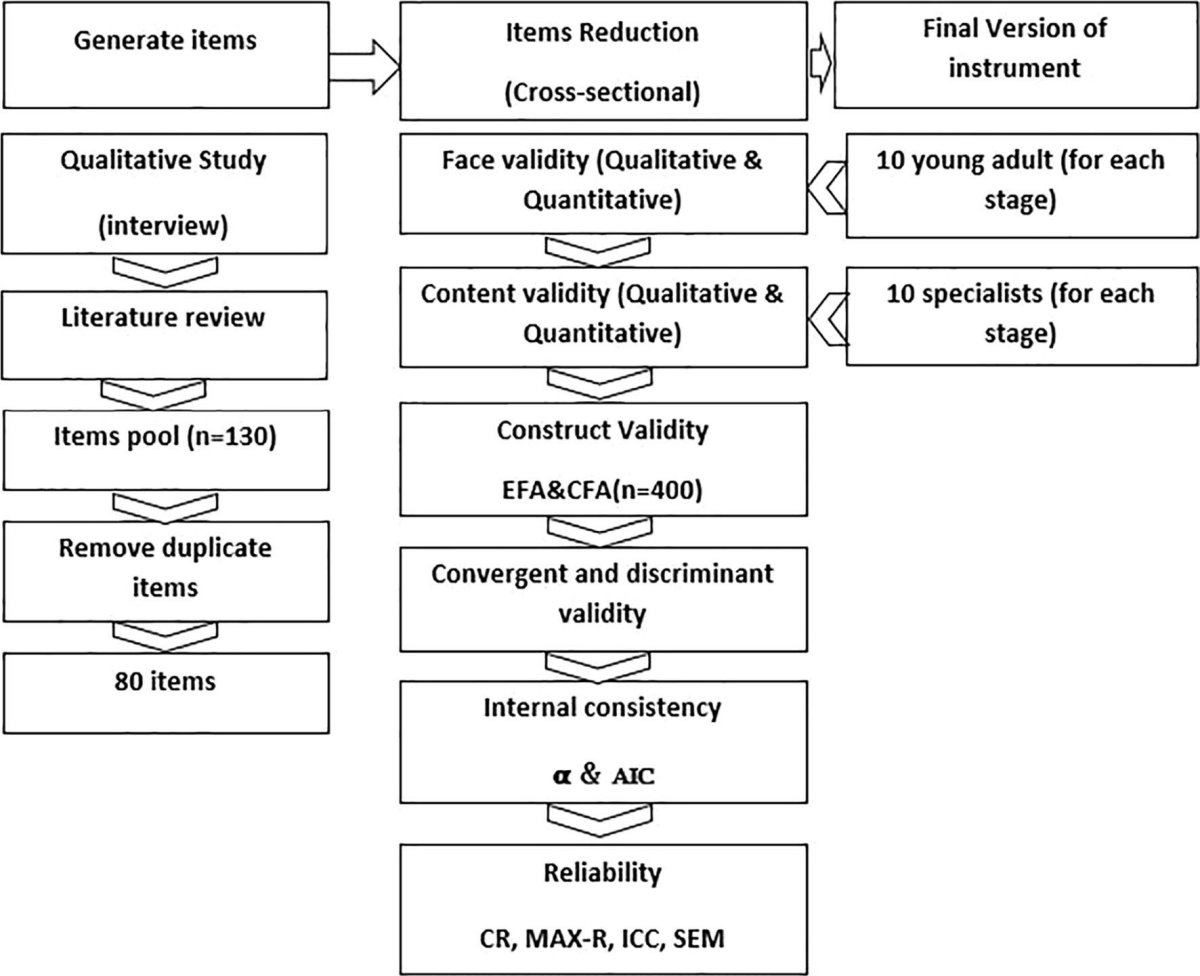
Flow chart of two phases of study

This exploratory sequential mixed—method study was conducted from May 2019 to March 2020 among young Iranian adults aged 18–26 years. It consisted of two steps, including (1) Generating items based on a qualitative study and literature review to form FI(HPV)VUBYA and (2) Reducing items by conducting a cross-sectional study for psychometric assessment of items obtained in the first step (Fig. 1).

## Methods

### Step 1: generating items

#### Qualitative study

An in-depth understanding of the concept was achieved using conventional content analysis and face-to-face, in-depth, and semi-structured interviews from May to September 2019. Thirty participants (10 young adults and 20 specialists) were invited for interviews. Participants were sampled purposefully with maximum variance (age, sex, marital status, education, occupation, and work experience). Interview questions were designed and two interviews were conducted as a pilot and minor changes were made to the questions. Interviews were then conducted by one of the authors (the duration of each interview was 30–60 min) and each interview was transcribed. The transcripts of the interviews were analyzed using MAXQDA10 software and Graneheim and Lundman method. Similar codes were placed in sub-categories and related sub-categories were placed in categories and named. Lincoln and Guba's criteria were used to ensure the reliability of quality data, which included credibility, dependability, confirmability, and transferability [37].

#### Literature review

In this study, by reviewing the existing texts and literature using keywords “Human Papillomavirus Vaccine, Effective Factors or Determinant, Barriers, Motivation, Facilitators, Young adults” in the databases “Science Direct, Pub Med, ProQuest, Scopus” and Persian databases, related articles from 2000 to 2019 were reviewed. Availability of the full text of the article in Persian and English was the inclusion criterion. In the initial search, 500 articles (480 English and 20 Persian languages) and 10 dissertations were obtained. After removing duplicates and irrelevant studies, 30 articles (20 English and 10 Persian languages) and 3 dissertations remained. Primary codes FI(HPV)VUBYA were extracted from them.

### Preparing the scale

Finally, FI(HPV)VUBYA were determined by using the codes extracted from the qualitative findings section and literature review. Based on the extracted codes, the appropriate expressions were placed in each categories and the total number of items was 130. In repeated sessions of the research team, writing, grammar, and similarities were examined, and some items were merged and deleted. Thus, the number of items was reduced from 130 to 90 items and to 80 items after re-examination. Finally, FI(HPV)VUBYAS with 80 items was prepared for psychometric assessment.

#### Step 2: reducing items

Features of FI(HPV)VUBYAS for knowledge items with a 3-point Likert scale (yes, no, and I do not know) and for the rest of the items (attitude) with a 5-point Likert scale (strongly agree to strongly disagree) in terms of face validity, content, construct, and reliability were evaluated.

### Face validity

Qualitative and quantitative face validity was used for FI(HPV)VUBYAS. Ten young adults evaluated the items. They were first asked to rate the scale based on the difficulty or ambiguity of the items. According to the participants, the necessary modification was made for some items. In quantitative face validity, the impact score of the items was calculated. The 10 young adults were asked to choose one of the following five answers for each items: “extremely important, important, average importance, of little importance, and not important at all” scores were determined based on the Likert scale (1–5 scores). Score 1 indicated the not important at all and score 5 indicated the extremely important. Using the (Impact Score = Frequency (%) × Importance) formula, items with an impact factor score greater than 1.5 were acceptable [38]. Fourteen items on the scale received an impact score of less than 1.5, which were removed according to the research team's opinion. The scale entered the content validity step with 66 items.

### Content validity

In qualitative content validity, 10 specialists in health education, reproductive health, psychologist, and social physician were used to review the items based on grammar, writing, and proper placement of items as well as appropriate scoring. According to their feedback, 10 items were removed and 2 items were added. Then, 58 items entered the quantitative content validity step. The content validity ratio (CVR) was examined to assess the necessity of the items (essential 3, useful but not essential 2, and not necessary 1). Given that the number of specialists was 10, the minimum acceptable CVR score based on Lawshe was considered to be 0.62 [39]. At this step, 4 items were removed (CVR < 0.62). Eventually, the items were reduced from 58 to 54. The Content Validity Index (CVI) showed how relevant each item is to the 4-point Likert scale (related 4, relevant but need minor revision 3, somewhat related but needs to be revised 2, and unrelated 1) conducted by 10 other specialists. All items were above 0.79. To eliminate the odds ratio, Kappa was calculated for each item (good = 0.60–0.74 and excellent value of Kappa > 0.74) [40]. All items had acceptable Kappa value. Also, 10 specialists independently evaluated the scale items. The agreement between the evaluators was calculated using the Intra-Class Correlation Coefficient (ICC) with the Two-Way Random model. Good agreement was reported between the evaluators (ICC = 0.83, *P* < 0.001).

### Item analysis

To identify possible problems with items before construct validity, items analysis was performed on 30 young adults, 60% of whom were female, 86.7% unvaccinated, and 56.7% aged 24–26 years. Pearson correlation coefficient was used to examine the correlation between items. No significant relationship was observed between items. Alpha Cronbach of the total items was 0.769, which is acceptable [40].

### Construct validity

The knowledge items (13 items) were based on literature review and were completely scientific and evidence-based. If EFA is not performed on these items, it will not reduce their validity, so they did not require EFA. The remaining attitude items (41 items) were entered into EFA and evaluated with the maximum likelihood and Promax rotation. Sample adequacy was estimated by Kaiser–Meyer–Olkin (KMO) and Bartlett tests. KMO 0.7–0.8 and 0.8–0.9 was interpreted well and excellent, respectively [41]. The sample size was estimated for factor analysis using a general rule considering 200 participants as the appropriate sample size [42]. In this study, two independent samples were collected, including 200 samples for EFA and 200 samples for CFA. A total of 400 young adults were included in the study. Demographic characteristics and HPV-related information of young adults are presented in Table 1.

**Table 1:** Demographic characteristics and information about HPV and HPV vaccination of participants (N = 400)

Variables	Sub-group	N (%)
Age	18–21	65 (16.3)
	21–24	98 (24.5)
	24–27	237 (59.2)
Gender	Female	272 (68.0)
	Male	128 (32.0)
Education	Sub—diploma or diploma degree	74 (18.4)
	Associate degree	41 (10.3)
	Bachelor degree	171 (42.8)
	Masters and PHD degree	114 (28.5)
Marital status	Single	294 (73.5)
	Married	91 (22.8)
	Widow/divorced	15 (3.7)
Job	Student	163 (40.8)
	Employee	88 (22.0)
	Self- employee	149 (37.2)
Income	Good income	32 (8.0)
	Median income	159 (39.7)
	Low income	73 (18.3)
	Not income	136 (34.0)
Do you have HPV?	Yes	72 (18.0)
	No	328 (82.0)
Did you uptake the HPV vaccine?	Yes	170 (42.5)
	No	230 (57.5)

Online data collection was done for this section. The online questionnaire was designed through the Porsline website and its URL link was sent to young adults aged 18–26 years through Telegram and WhatsApp channels. Data in Spss file were extracted from Porsline website and prepared for analysis. In order to select the appropriate variable and contribute to the formation of factors, percentage subscription equal to or greater than 0.4 were used [43]. To evaluate the structural factors, CFA was performed using the maximum likelihood method and the most common goodness-of-fit indices. The model fitness was assessed according to Parsimonious Normed Fit Index (PNFI), Root Means Square of Error of Approximation (RMSEA), Tucker–Lewis index (TLI), Incremental Fit Index (IFI), Comparative Fit Index (CFI), Parsimonious Comparative Fit Index (PCFI), and CMIN/DF.

### Convergent and discriminant validity

The convergent and discriminant validity of the extracted factors were estimated to Composite Reliability (CR) and Average Variance Extracted (AVE). To establish convergent validity: (a) CR should be greater than AVE and (b) AVE should be > 0.5. To meet the discriminant validity, AVE should be > 0.5 [44].

### Reliability

To evaluate the internal stability of FI(HPV)VUBYAS, Cronbach’s alpha (α) and Average Inter-item Correlation (AIC) were used, and AIC (0.2–0.4) was considered an acceptable internal consistency [45]. Then Composite Reliability (CR) and Max H reliability, which replaces Cronbach’s alpha coefficient in the SEM, were evaluated and > 0.7 value was considered acceptable [46]. The stability of FI(HPV)VUBYAS over time (test–retest) was measured using the Intraclass Correlation Coefficient (ICC) [47]. It was performed in two stages with 2 weeks’ intervals in 30 young adults. This time interval causes, on the one hand, the scale terms to be forgotten, and on the other hand, the phenomenon being measured to not be changed and values of 0.75 were acceptable [48].

### Normal distribution of data, outlier data, and missing data

Before CFA, it is necessary to perform CFA assumptions, including data normality and reviewing outlier data. Given the item scores are based on the Likert scale, skewness and kurtosis coefficients were used for the normality test. The test results showed that the skewness and kurtosis scores are in the range (− 2, + 2) and have a normal distribution. Regarding outlier data, when the constructs do not have a normal distribution there are outlier data, so when the scores of all constructs have a normal distribution, there is no outlier data to examine [49, 50].

#### Ethical considerations

The present study was approved by the Ethics Committee of the Neuroscience Research Center of Shahid Beheshti University of Medical Sciences with the code: IR.SBMU.PHNS.REC.1397.058. Before starting the study, the general objectives were explained to the participants and informed consent was obtained from them to enter the study. All the participants, both at the interview stage and while completing the scale, were assured that their information would remain confidential.

## Results

### Generating items

Based on the combination of the results of the qualitative study and literature review, 1100 raw codes were extracted. In the data analysis process, after reviewing and deleting irrelevant codes, 500 primary codes were extracted. After repeated reviews, merging codes, and classifying them, 150 main codes were obtained. After reviewing, due to the great similarity between the codes, they were classified into 66 main codes, which were placed in 16 sub-categories and 8 categories. Categories included knowledge, outcome expectations, perceived threat, external stimuli, environment, responsibility, perceived barriers, and perceived stigma.

### Reducing items

After performing face and content validity, the scale items were reduced from 80 to 54 (13 knowledge items and 41 attitude items). In EFA, the KMO test value was 0.875 and the Bartlett test value was 5523.990 (*P* < 0.001). Seven factors (38 items) were extracted and titled “external stimuli” (7 items), “perceived vulnerability” (6 items), “perceived stigma” (6 items), “perceived severity” (6 items), “outcome expectations” (4 items), “environment” (4 items), and “perceived barriers” (5 items). These 7 factors explained 57.84% of the total variance of FI(HPV)VUBYA (Table 2). Cronbach's alpha coefficient for the whole scale was calculated at 0.86 and for each factor was from 0.7 to 0.91. The scale ICC was 0.90, indicating the optimal stability of the scale (Table 3).

**Table 2:** the seven factors of FI(HPV)VUBYA and their factor loadings (N = 200)

Factors	Items	Factor Loading	h^2^	*λ*	Variance (%)
External stimuli	57. If friends suggest (or have suggested) the HPV vaccination, I will inject (or have injected)	0.952	0.778	11.487	28.016
	54. If my family members suggest (or have suggested) the HPV vaccination, I will inject (or have injected)	0.867	0.709		
	60. If my family members inject the HPV vaccine (Or they injected), I inject (or injected)	0.843	0.693		
	55. If my partner (or my future partner) recommends (or has recommended) the HPV vaccine, I will inject (or have injected) it	0.813	0.749		
	59. If my friends inject the HPV vaccine (Or they injected it), I inject it (or injected it)	0.756	0.543		
	56. If your doctor recommends (or has recommended) the HPV vaccine, I will inject (or have injected)	0.727	0.684		
	58. If celebrities (clergy, athletes …) emphasize the importance of injecting the HPV vaccine, I will inject (or inject)	0.621	0.433		
Perceived vulnerability	24. I feel like I'm currently at risk for cancer (cervix, urethra, vagina, mouth, and throat)	0.922	0.745	4.035	9.842
	26. I feel like I'm at risk for genital warts right now	0.880	0.706		
	25. I feel I will be exposed to genital warts in the future	0.739	0.737		
	28. I feel like I'm currently exposed to HPV through my sexual partner	0.719	0.578		
	23. I feel I may be at risk for cancer (cervix, penis, anus, mouth) in the future	0.637	0.594		
	27. I feel I will be exposed to HPV through my sexual partner in the future	0.619	0.644		
Perceived stigma	51. The person receiving the HPV vaccine is ridiculed by their friends and acquaintances to her/him	0.899	0.831	2.814	6.864
	50. The person receiving the HPV vaccine receives the social stigma of immorality	0.879	0.767		
	52. Fear of potential criticism from others causes the HPV vaccine to be avoided	0.813	0.690		
	44. The HPV vaccine is not injected because people feel shy about diseases caused by HPV	0.521	0.338		
	53. There is some doubt about the HPV vaccine because the mass media (TV, newspapers, cyberspace) present a negative image of the HPV vaccine	0.496	0.320		
	42. Improper behavior of healthcare professionals or healthcare is one of the reasons for not coming for the HPV vaccination	0.412	0.380		
Perceived severity	32. If I get HPV-related diseases, I will have family problems	0.974	0.609	1.750	4.268
	31. Having an HPV-related disease will damage my social relationships with others	0.945	0.635		
	33. Having an HPV-related disease will hurt my sexual relationship	0.752	0.644		
	30. If I get cancer (cervix, urinary tract, anus, mouth, and throat), I will have long-term problems with the diseases	0.646	0.652		
	29. If I get genital warts, it is a serious problem for my health	0.508	0.640		
	34. If I get high-risk HPV, it can be fatal in the long run	0.477	0.439		
Outcome expectations	14. If I inject (or have injected) the HPV vaccine, it will help prevent disease (cancers and genital warts)	0.851	0.584	1.246	3.038
	15. If I inject (or have injected) the HPV vaccine, the Stress caused the HPV-related diseases (cancers and genital warts) is reduced	0.809	0.649		
	16. If I inject (or have injected) the HPV vaccine, it will reduce deaths from HPV-related diseases (cancers)	0.789	0.656		
	17. If I inject (or have injected) the HPV vaccine, it will be very helpful for me	0.633	0.654		
Environment	37. There are no media training programs (TV, cyberspace) to inform and encourage the importance of HPV vaccination	− 0.941	0.673	1.246	3.040
	38. There are no training programs in health centers and hospitals to inform and encourage the importance of HPV vaccination	− 0.806	0.612		
	39. There are no training programs in schools and universities to inform and encourage the importance of HPV vaccination	− 0.766	0.522		
	35. Difficult to obtain the HPV vaccine in pharmacies	0.432	0.178		
Perceived barriers	19. If I inject (or have injected) the HPV vaccine, it will cause a lot of pain at the site of the injection	0.725	0.497	1.138	2.777
	40. It takes time to Inject three—doses of the HPV vaccine	0.648	0.302		
	20. If I inject (or have injected) the HPV vaccine, it may cause nausea	0.623	0.423		
	36. It takes a long way to go to hospitals and health centers to get vaccinated	0.552	0.290		
	41. The HPV vaccine is expensive to inject	0.464	0.187		

h^2^, item communality, λ, eigenvalue

**Table 3:** The Cronbach’s alpha and the ICC for FI(HPV)VUBYA

Factors	Number of items	Cronbach’s alpha	Interclass correlation coefficient (95% CI)
External stimuli	7	0.91	0.91 (0.86–0.95)
Perceived Vulnerability	6	0.89	0.89 (0.82–0.94)
Perceived severity	6	0.88	0.88 (0.81–0.93)
Perceived stigma	6	0.81	0.88 (0.81–0.93)
Outcome Expectations	4	0.85	0.85 (0.76–0.92)
Eenvironment	4	0.88	0.88 (0.81–0.93)
Perceived barriers	5	0.78	0.78 (0.64–0.88)
Knowledge	13	0.84	0.84 (0.75–0.91)
Total	51	0.86	0.90 (0.84–0.94)

*ICC* intra-class correlation coefficient

**Fig. 2:**
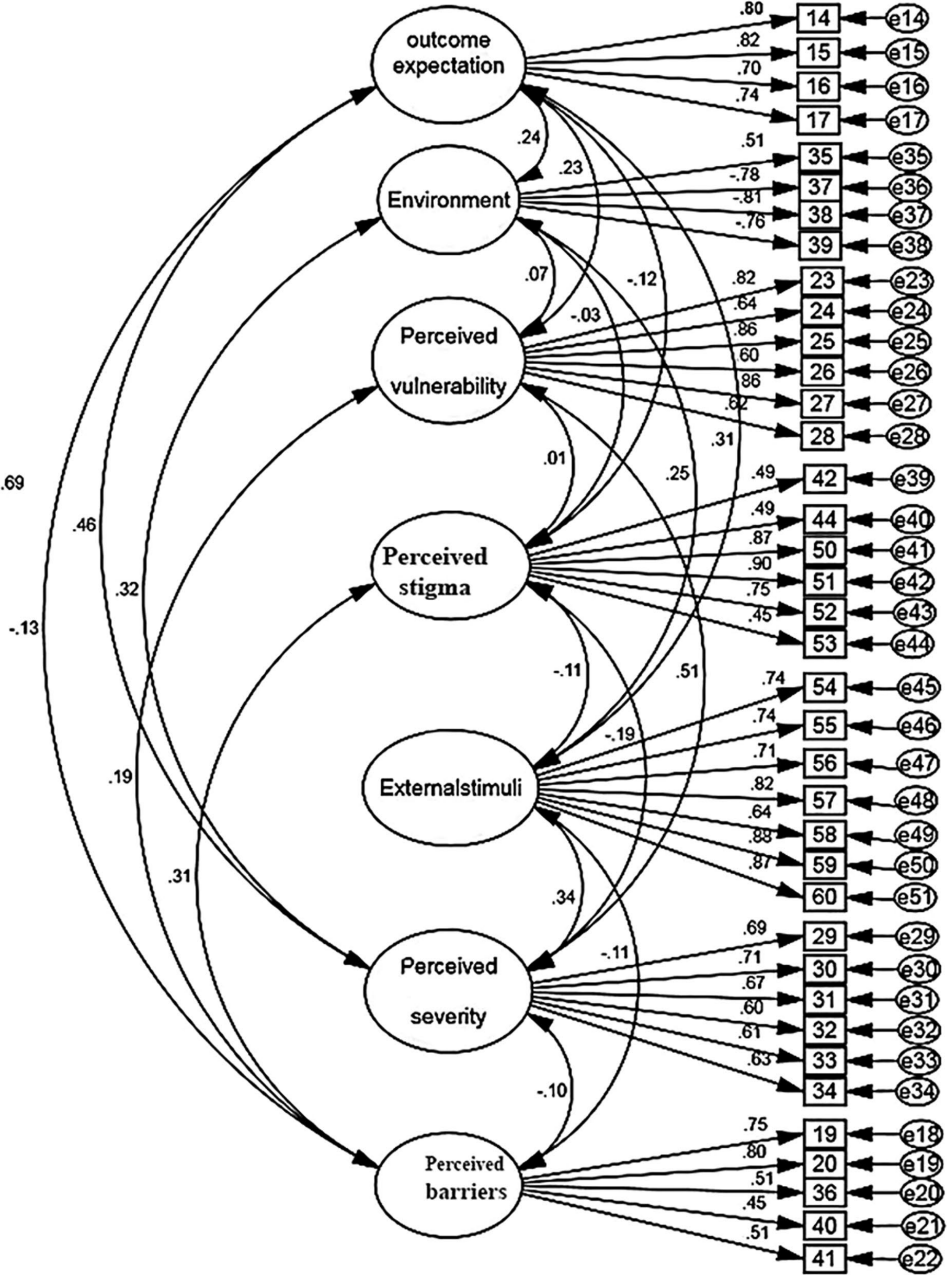
The FI (HPV)VUBYAS construct: a model of first-order confirmatory factor analysis (N = 200)

**Fig. 3:**
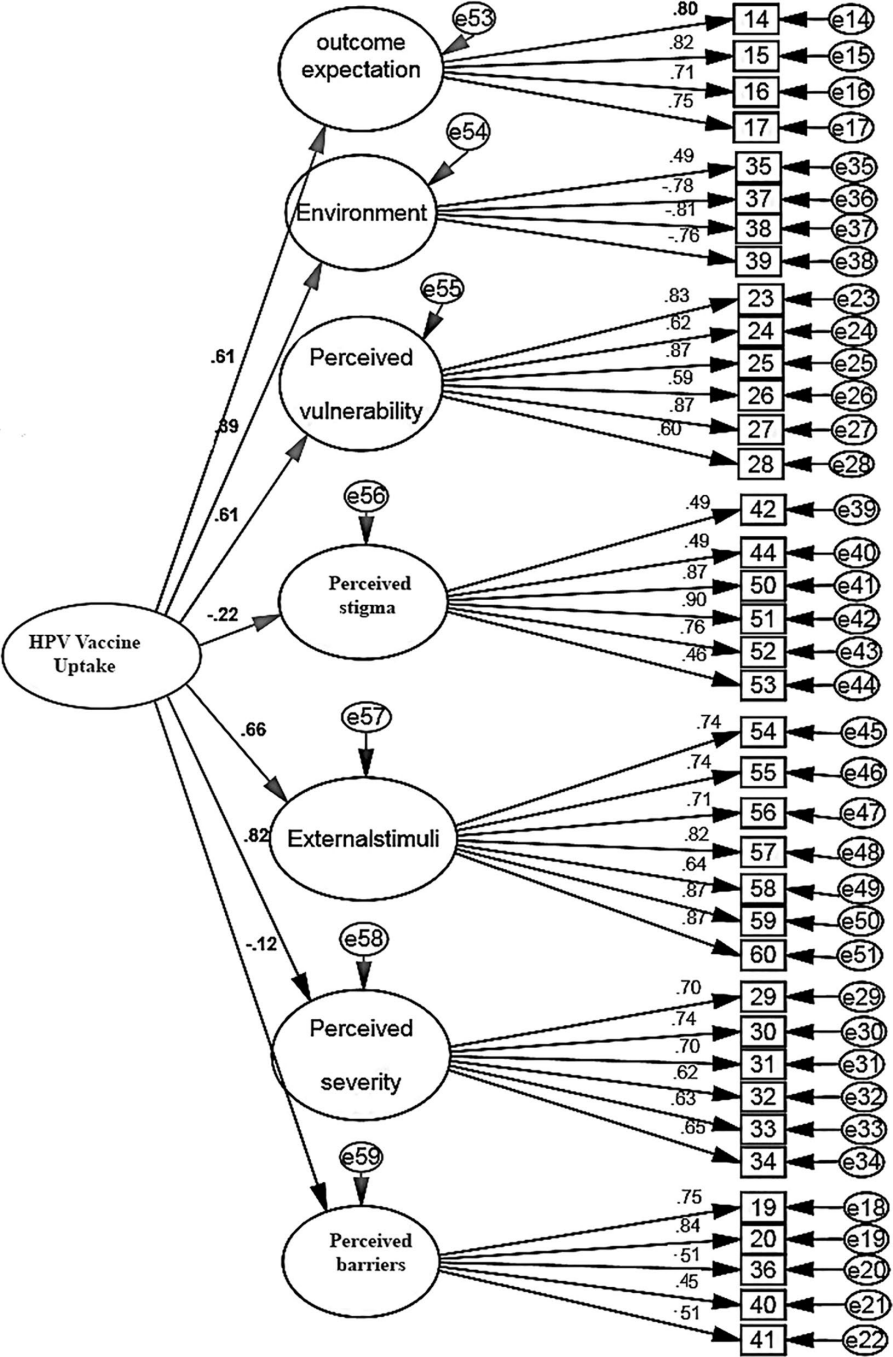
The FI (HPV)VUBYAS construct: a model of second-order confirmatory factor analysis (N = 200)

In the CFA, the chi-square fit index of 1555.056 (*P* < 0.001) and CMIN/DF = 2.415 were calculated. Other model fit indices were calculated and these good model fit values confirmed the final model (Table 4 and Fig. 2). The second-factor analysis was performed to confirm the latent variable of vaccination behavior. Figure 3 reveals the second-order structural model and CFA. The results of AVE, CR, and Cronbach's alpha confirmed that the second CFA model has convergent and divergent validity (Table 5). The internal consistency of the scale showed that Cronbach's alpha and ALC of all factors are greater than 0.7 and 0.5, respectively. Moreover, CR and Max H reliability of factors showed that there is a strong coefficient (Table 5). The SEM value for the scale was 6.78%, indicating that the individuals' scores on this scale tend to distribute 6.78 values around their "correct" score.

**Table 4:** Fit indices of the confirmatory factor analysis of the FI(HPV)VUBYAS (N = 200)

Indices	*Χ*^*2*^	df	*P*-value	CMIN/DF	RMSEA	PNFI	PCFI	TLI	IFI	CFI
First-order after structure modification	1555.056	644	0.000	2.415	0.084	0.615	0.709	0.953	0.977	0.974

Fitness indexes: PNFI, PCFI (> 0.5); TLI, IFI, CFI (> 0.9), RMSEA (< 0.08), CMIN/DF (< 3 good, < 5 acceptable)

*CFI* comparative fit index, *CMIN/DF* Minimum discrepancy function divided by degrees of freedom, *df*, degree of freedom, *IFI* incremental fit index, *PCFI* parsimonious comparative fit index, *PNFI* parsimonious normed fit index, *RMSEA* root mean square error of approximation, *TLI* Tuker–Lewis index

**Table 5:** The indices of the convergent, discriminant validity and internal consistency of FI(HPV)VUBYAS for CFA(N = 200)

Factors	Indices				
	AVE	CR	MaxR(H)	Alpha	AIC
Outcome expectations	0.690	0.899	0.901	0853	0.663
Perceived barriers	0.827	0.905	0.796	0.791	0.801
Perceived vulnerability	0.830	0.936	0.920	0897	0.805
Perceived severity	0.532	0.846	0.944	0.807	0.510
Environment	0.650	0.844	0.853	0829	0.590
Perceived stigma	0.566	0.863	0.886	0.813	0.532
External stimuli	0.656	0.930	0.922	0912	0.636

*AVE* average variance extracted, *CR* composite reliability, *MaxR* (*H*) maximum reliability; Alpha, Cronbach’s alpha, *AIC* average inter-item correlation

## Discussion

The results of this study showed that FI(HPV)VUBYAS had good validity and reliability and included 51 items and 8 factors (knowledge, external stimuli, perceived vulnerability, perceived stigma, perceived severity, outcome expectations, environment, and perceived barriers), explaining 57.84% of the total extracted variance. CFA was used in this study and confirmed the fit of the FI(HPV)VUBYAS model.

The purpose of factor extraction was to maximize variance [51], which was 57.84% in this study. The highest amount of variance was related to the external stimulus (28.01%) and perceived vulnerability (9.84%), respectively. In the study of Ali et al. (2017), which was somewhat similar to the present study, EFA was not used and no variance was reported [32]. In the study by Thomas et al. [33], psychometric assessment explained students’ opinions about HPV and its vaccine with 4 factors and 40% of the total variance [33]. Guvenc et al. [52] also reported the HPV health belief model scale and its vaccination in nursing students with 4 factors and 61.47% of the total variance [52].

According to Cronbach's alpha results, FI(HPV)VUBYAS showed strong and excellent internal consistency. FI(HPV)VUBYAS also has strong stability with an acceptable value of ICC, which is one of the advantages of this scale. In this study, the SEM scale was calculated. Measurement error is an important and required domain of COSMIN (Consensus-based standards for the selection of health Measurement Instruments) [53]. A smaller amount of SEM is very important on a scale. In fact, SEM determines the accuracy of each person's score. Previous psychometric assessment studies did not report the scale for evaluating HPV factors for this index.

Based on the factor load of the items in FI(HPV)VUBYA, 7 extracted factors (external stimuli, perceived vulnerability, perceived stigma, perceived severity, outcome expectations, environment, and perceived barriers), the first extracted factor was external stimuli. This factor consisted of 7 items reflecting the effect of others and the media on HPV vaccination in young adults. Among all the factors, external stimuli had the greatest effect on getting the vaccine. In the study by Peterson et al. [54], the effect of health care providers, peers, and social support were reported as effective factors on vaccination [54]. Emerson et al. [55] reported that parental satisfaction was effective in adolescent vaccination [55]. The second extracted factor was the 6 items perceived vulnerability, providing young adults an understanding of how they are at risk for HPV disease and cancer. These studies have shown that vulnerability to the disease plays a role in the intention of getting a vaccination [56, 57]. Several scales of HPV health belief model and HPV vaccine had this factor [33, 52]. It is important to note that although the names of the factors were similar at different scales for the HPV vaccine, they were different in terms of items.

The third factor was the perceived stigma and 6 items represented a negative association between the community, others, and health care providers with the person getting the vaccine. In a study by Jones et al. [58], it was emphasized that ethnicity and place of residence significantly affect HPV-related stigma. This was mostly reported in men who had the HPV vaccine available [58]. No scale was found to examine perceived stigma as a separate factor.

Perceived severity was the fourth extracted factor with 6 items, indicating the perceived severity of risk and damage caused by HPV in young adults, which encourage or prevent them from getting the HPV vaccine. Several scales of the HPV health belief model and the HPV vaccine reported this factor [33, 52]. However, in these scales, their perceived severity factor was different from the present study in terms of questions. In the study by Christy et al. [59], women and men who did not get the vaccine reported their perceived severity of HPV-related diseases, and their regret levels for not getting the vaccine were reported to be high [59].

The fifth extracted factor was the outcome expectations with 4 items, indicating the benefits of the young adult getting the vaccine and also the possible results of vaccination. No such a factor was reported on any scale. Thompson et al. [60] stated that the outcome expectations factor is one of the effective factors of parents on their children's HPV vaccination [60]. The sixth extracted factor was the environment with 4 items, representing the physical or social conditions of individuals, including providing educational opportunities to overcome personal and situational barriers or providing access to health services, such as vaccines. No such a factor was found in any of the scales examining HPV vaccination. Yarmohammadi et al. [61], reported education and access to vaccines as important strategies for encouraging young adults to uptake the HPV vaccine [61].

The last factor was perceived barriers with 5 items related to barriers, such as the vaccine costs, the vaccine side effects, the long intervals between three doses of the vaccine, and the long-distance from health centers. Although these are basic vaccination requirements, they are likely to affect vaccination as well. Therefore, extensive research studies have been conducted in this field [20, 62].

## Limitations

The study of young adult subjects was selected from Tehran, so generalizability could be limited. The scales were also completed online and this can somewhat reduce the communication with the participants as well as the accuracy of their responses.

## Conclusion

The results of this study show that FI(HPV)VUBYAS consisted of 51 items with 8 factors has acceptable validity and reliability. Based on the findings of this study, knowledge, external stimuli, perceived vulnerability, perceived stigma, perceived severity, outcome expectations, environment, and perceived barriers can be used to provide HPV vaccination services.

## Data Availability

The dataset generated and analyzed during the current study is not publicly available due to the highly sensitive nature of interview transcript data. Publication of entire transcripts risks identifying research participants. Data used in this study is analyzed and the data is available from the corresponding author upon reasonable request.

## Abbreviations

FI(HPV)VUBYA: Factors influencing HPV vaccine uptake behaviors in young adults
FI(HPV)VUBYAS: Factors influencing HPV vaccine uptake behaviors in young adults scale
EFA: Exploratory factor analysis
CFA: Confirmatory factor analysis
HPV: Human papillomavirus
CVR: Content validity ratio
CVI: Content validity index
ICC: Intra-class correlation coefficient
KMO: Kaiser–Meyer–Olkin
CR: Composite reliability
